# Virtual surgical planning guided osteotomy in facial feminization surgery

**DOI:** 10.1016/j.jpra.2026.05.039

**Published:** 2026-06-03

**Authors:** Hester Elizabeth Lacey, Yi Min Khoong, Keith Altman

**Affiliations:** a Flinders University, Registry Rd, Bedford Park, SA 5042, Australia; b Brighton and Sussex Medical School, University of Sussex, Falmer, BN1 9PH, United Kingdom; c Department of Oral & Maxillofacial Surgery, Queen Elizabeth University Hospital, NHS Greater Glasgow & Clyde, 1345 Govan Rd, Glasgow G51 4TF, United Kingdom; d Queen Victoria Hospital, Holtye Road, East Grinstead, West Sussex RH19 3DZ, United Kingdom

**Keywords:** Virtual surgical planning, Facial feminization surgery, Osteotomy

## Abstract

**Background:**

Osseous facial feminization surgery (FFS) requires precision to accurately modify craniofacial skeletal features and achieve optimal aesthetic and functional outcomes. Virtual surgical planning (VSP) has emerged as a tool to enhance preoperative planning and intraoperative accuracy in FFS. This systematic review aims to evaluate the role of VSP in guiding osteotomy during osseous FFS, assessing operative efficiency, accuracy, safety, and satisfaction, and describe potential future applications.

**Methods:**

A systematic review was conducted in accordance with PRISMA guidelines and the Cochrane Handbook. MEDLINE, EMBASE, Global Health, Scopus, Web of Science, Cochrane Central Library, and grey literature sources were searched in April 2025. Eligible studies included adult transgender women undergoing osseous FFS procedures with VSP guidance. Outcomes assessed included planning time, operative time, accuracy, safety/complication rates, and patient-reported satisfaction or femininity perception. Meta-analysis was performed for operative time using standardised mean difference (SMD).

**Results:**

Seven studies met inclusion criteria. Meta-analysis demonstrated significantly reduced operative time with VSP compared to conventional techniques (SMD = −0.72; 95 % CI −1.13 to −0.32; P < 0.05). Planning time varied substantially across studies reporting this data (5–10, 145 ± 13.2 min), reflecting heterogeneity in outcome reporting, software platforms used and workflow integration. Accuracy outcomes favoured VSP, with one comparative study reporting 95.25 ± 4.09 % accuracy with VSP versus 78.25 ± 12.92 % without. Reported complication rates were low (0–1.77 %), though comparative safety data were limited. Patient satisfaction and cosmetic outcomes were consistently high, though assessed using heterogeneous tools precluding pooled analysis.

**Conclusions:**

Current evidence suggests that VSP in osseous FFS is associated with reduced operative time, improved osteotomy accuracy, and favourable safety and patient-reported outcomes. Although methodological heterogeneity and limited comparative data constrain definitive conclusions, findings support an expanding role for VSP in precision-driven craniofacial gender-affirming surgery. Emerging integration of augmented reality (AR)-assisted intraoperative navigation may further enhance anatomical accuracy and surgical safety. Prospective comparative studies with standardised outcome measures and validated patient-reported instruments are required to define the long-term clinical and economic value of VSP and AR in FFS.

## Introduction

### Rationale

Virtual surgical planning (VSP) describes the use of high-resolution cross-sectional imaging with computer assisted 3D reconstruction to facilitate digital analyses of patient anatomy, used in surgical specialities to simulate and plan surgical steps and navigation of patient anatomy, prior to entering the operating theatre.^1^ VSP is increasingly predominant in surgical subspecialities where precise and predictable, steps and navigation is essential to produce optimal surgical outcomes. Digital analysis of patient anatomy and 3D reconstruction allows surgeons to familiarise themselves closely with the pertinent patient anatomy and produce detailed plans of bone and soft tissue navigation to precisely plan operative steps.^2^

Facial feminization surgery (FFS) describes a range of soft tissue and osseus procedures used in facial surgery to modify the stereotypical masculine facial features to produce a more aesthetically feminine appearance.^3^ FFS is commonly used in the field of gender affirmation surgery, to align the appearance of a transgender woman more closely with their lived experience of their gender, to alleviate gender dysphoria, reduced gender incongruence, and improve health related quality of life.^4^ A range of FFS procedures have been described, including reconstructive cranioplasty, genioplasty, mandibular angle reduction, hairline advancement, brow lift, rhinoplasty, cheek and lip augmentation and thyroid shave.^5^

Osseous craniofacial procedures, involving precisely controlled, segmentalized bony osteotomy to reshape or reconstruct the bony structures of the skull, face, and jaws, described in combination as reconstructive cranioplasty, form a cornerstone of FFS procedures.^6^ The most frequently performed osseous craniofacial procedures include setback of the anterior frontal sinus wall, lateral supraorbital recontouring, osseous genioplasty, and mandibular angle reduction. Osseous FFS procedures involve precision osteotomy to modify the aesthetic appearance of the skeletal facies, with millimetres level differences significant in influencing the final aesthetic and functional results, and subjective patient satisfaction with outcome.^7^

In osseous FFS, VSP is increasingly recognised for the role it can play in facilitating highly accurate and data driven preoperative planning and intraoperative precision.^8^ VSP aids pre-osteotomy planning and execution by facilitating pre-operative analysis of bone morphology, planning of osteotomy depth, angle and contour, and simulating bone repositioning and modified soft tissue relations.^9^ VSP allows creation of custom cutting guides, plates and implants to facilitate optimization of intraoperative surgical steps and highest post-operative patient satisfaction.^10^ This is especially valuable in complex or revision cases, or where the anatomy is complex or variable, offering enhanced intraoperative anatomical safety, by mapping osteotomy boundaries and reducing the risk of dural exposure, breach and CSF leak.^9^

This review aims to establish the role of VSP in osseous craniofacial FFS, to assess outcomes, benefits and any associated drawbacks or complications with use, and relevant financial costs.

## Methods

### Eligibility criteria

Studies eligible for inclusion were randomised controlled trials, systematic reviews, cohort studies, case-control studies and case series, case reports.

Exclusion criteria were the following: animal studies, experimental studies, in vitro studies, letters to editor and expert opinions, non-English language studies, and articles where no full text was available.

### Search strategy and information sources

Databases searched included the MEDLINE, EMBASE, Global Health, Scopus and Web of Science databases, and Cochrane Central Library. Google scholar was used to search for grey literature. The references of included studies were screened to identify further papers relevant to the review. The search was executed in April 2025. The full search strategy is included in Appendix A.

### Selection process

Title and abstract screening and full-text review were performed by two separate reviewers with reference to the study inclusion/exclusion criteria. Conflicts were resolved by a third, senior reviewer.

### Data collection process

Data collection was performed using a bespoke data collection tool on the Covidence platform.^11^ Two reviewers collected data separately, with conflicts resolved by a third, senior reviewer.

### Data items

Extracted data included study characteristics (e.g., country, study design, sample size, inclusion criteria), patients’ demographic data (e.g., age, facial regions, types of FFS procedures), technology used (e.g., VSP method), and outcomes (e.g., planning time, operative time, safety or complication rate, accuracy, patients’ satisfaction, femininity perception score, cosmetic improvement).

### Study risk of bias assessment

Risk of bias assessment was performed using the Robins risk of bias assessment tool. Two separate reviewers performed a risk of bias assessment for each study, with conflicts resolved by a third, senior reviewer.

### Effect measures

The population of interest included adults (>18) undergoing facial feminization procedures. The intervention studied was the use of VSP in FFS procedures involving cranial osteotomy, including^1^ setback of the anterior frontal sinus wall,^2^ lateral supraorbital recontouring,^3^ osseous genioplasty,^4^ mandibular angle reduction. The comparator was FFS performed without the use of VSP.

Meta-analyses of primary outcomes were conducted in RevMan 5.4.1, using appropriate effect measures based on the type of outcome.^12^ To compare the efficiency of VSP (experimental group) with non-VSP (control group), 2-arm meta-analysis was performed. For continuous outcomes, standardised mean difference (SMD) was used when measuring units that varied across studies.

### Synthesis methods

When studies reported outcomes separately by facial region or procedure type, subgroup data were combined using standard methods recommended in the Cochrane Handbook to obtain a single mean and standard deviation for inclusion in the meta-analysis^13^

Meta-analyses were performed using either a random effects model or a fixed effects model based on the heterogeneity among studies. If I2 ≥ 50 %, the data were considered statistically heterogeneous, and a random effects model was used for data analysis.

## Results

### Study selection

**Fig. 1:**
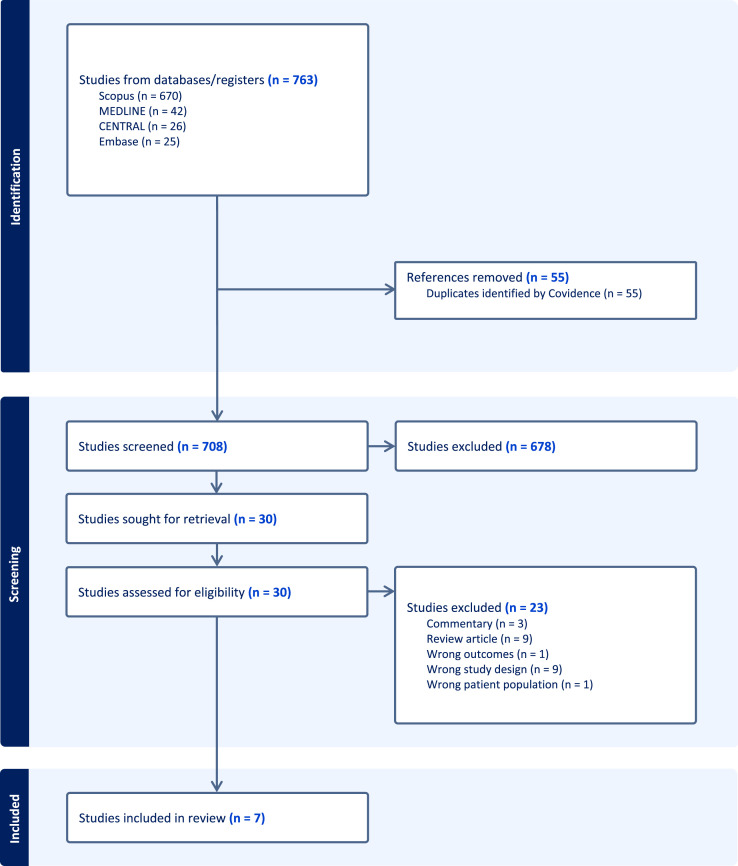
PRISMA study selection diagram.

A total of 763 articles were identified, of which 55 were duplicates. Of the remaining studies, 677 were excluded after title and abstract screening and a further 23 after full-text review. With further full text reference and review of relevant articles from references, a total of 7 studies met the inclusion and exclusion criteria for this systematic review. An overview of the search results is presented in the PRISMA flowchart in Fig. 1.

### Risk of bias in studies

The risk of bias for included studies was assessed using the ROBINs-I Risk of Bias in Non-randomised Studies of Interventions Tool and presented using the Robvis visualisation tool.^14^ Overall, there was a moderate risk of bias in 6 of the 7 included studies. The most common area of bias was bias due to confounding, where is most studies, a lack of comprehensive reporting of methodological factors or considerations which may introduce bias meant that full assessment of potential confounding variables could not take place. A funnel plot was not generated due to heterogeneity in study designs and outcomes among the included studies, which would limit its interpretability. The full risk of bias assessment is included in Appendix B.

## Results of individual studies

### Study demographics

Characteristics of the included studies are provided in Table 1.^15^, ^16^, ^17^, ^18^, ^19^, ^20^, ^21^ Patient demographic data are provided in Table 2.

**Table 1:** Characteristics of included studies.

Study	Country	Study design	VSP method	Inclusion criteria
Ganry and Cömert, 2022^15^	France	Experimental	Homemade VSP open-source software protocol using a MacBook Pro with 4 open-source software (OsiriX, Meshlab, Nettfab, Blender)	Patient after a minimum of 6 months following frontal sinus anterior wall setback
Gray et al., 2019^16^	United States	Comparative cadaveric studies	VSP and CAD/CAM	Male cadaveric heads underwent morphologic typing analysis of the frontal brow, lateral brow, mandibular angle, and chin regions
Gursky et al., 2025^17^	United States	Retrospective cohort study	Not mentioned	Transfeminine women or gender-diverse individuals who underwent FFS
Gutierrez-Santamaría et al., 2024^18^	Spain	Prospective case series	Software developed in conjunction with Nemotec (Biotech Dental, Madrid, Spain)	Transfeminine patients who underwent FFS
Hu et al., 2025^19^	United States	Retrospective cohort study	VSP for osteotomy (with engineers (Materialise) to create an anticipated final skull 3D reconstruction)	Patients who underwent genioplasty, mandibular contouring, or both with a single surgeon
LaPadula et al., 2019^20^	France	Retrospective cohort study	CAD softwares (Meshmixer® and Blender), followed by Suite® to set up the printing	Patients over 18 years of age; persistent and well-documented gender dysphoria, patients living at least 12 months in the gender role that is congruent with their gender identity, ability to make decisions and consent to a treatment, and patient under hormonal therapy since 12 consecutive months or more
Tawa et al., 2021^21^	France	Prospective cohort study	Based on CT DICOM, Virtual 3D models created are imported into ProPlan CMF 3.0 (Materialise, Leuven) for surgical planning. Cutting guide designed with Materialise engineering team utilizing 3-matic software (Materialise, Leuven, Belgium)	Type III frontal prominence according to the Ousterhout, acute mandibular angles with indication for bilateral resection, and wide and thick chin with indication for vertical resection

VSP: Virtual Surgical Planning; CAD: Computer-aided design; CAM: Computer-aided manufacturing.

**Table 2:** Patient demographic data.

Study	Age (Mean ± SD) (Range)	Sample size	Procedure	Facial regions
Ganry and Cömert, 2022^15^	30.75	16	Setback of the anterior frontal sinus wall.	Forehead, eyebrow
Gray et al., 2019^16^	54.3 ± 4	50 (25 each group)	1. Setback of the anterior frontal sinus wall. 2. Lateral supraorbital recontouring. 3. Osseous genioplasty. 4. Mandibular angle reduction.	Forehead, lateral brow, mandibular angle, chin
Gursky et al., 2025^17^	33.8 ± 9.58	226	Osseous genioplasty	Chin
Gutierrez-Santamaría et al., 2024^18^	33.2	30	1. 21 angle-to-angle ostectomies (3 with chin advancement). 2. 5 chin and mandibular body ostectomies (3 with chin advancement). 3. 4 chin ostectomies.	Mandibular angle, mandibular body, chin
Hu et al., 2025^19^	Not mentioned	14	1. 3-piece genioplasty. 2. Genioplasty with inferior border resection. 3. VSP-guided mandibular osteotomy. 4. Freehand mandibular contouring.	Mandible including chin
LaPadula et al., 2019^20^	Not mentioned	25	1. Upper third of the face: frontal bone grinding, hairline advancement. 2. Middle-third of the face: rhinoplasty, malar implants, malar valgus osteotomy, facial lipofilling. 3. Lower third of the face: grinding of the angles and mandibular rims, reduction of the masseter muscle, angle implants, upper lip lifting, orthognathic surgery, genioplasty, subchin liposuction, and reduction laryngoplasty.	Upper third, middle third and lower third of the face
Tawa et al., 2021^21^	31.3 (19–49)	45	1. Setback of the anterior frontal sinus wall. 2. Osseous genioplasty. 3. Mandibular angle reduction.	Forehead, mandibular angle, chin

### Results of synthesis

Due to the limited number of studies with control groups and inconsistent outcome reporting, meta-analysis was performed only for operative time. Table 3 summarizes results of included studies which reported outcomes of interest for this review.

**Table 3:** Results of all outcomes.

Study	Planning time (minutes)	Operative time (minutes)	Accuracy (%)	Safety (%)	Complication (%)	Satisfaction score	Cosmetic improvement/ Femininity Perception
Ganry and Cömert, 2022^15^	5 to 10						92 %
Gray et al., 2019^16^		With VSP: 22.5 ± 7.28 Without VSP: 31.25 ± 10.36	With VSP: 95.25 ± 4.09 Without VSP: 78.25 ± 12.92	With VSP: 100 Without VSP: 91			
Gursky et al., 2025^17^					1.77		
Gutierrez-Santamaría et al., 2024^18^		With VSP: 96.94 ± 29.60 Without VSP: 115.09 ± 40.19				8.14 ± 1.57	73.87 ± 15.37
LaPadula et al., 2019^20^	145 ± 13.2	420 ± 23			0	32.3 ± 6.9	
Tawa et al., 2021^21^			89.93 ± 8.6			94.40 %	

VSP: Virtual Surgical Planning.

### Planning time

Two studies reported planning time, ranging from 5 to 10 min in one study to 145 ± 13.2 min in the other. Meta-analysis was not performed due to the absence of comparator groups and substantial heterogeneity.

### Operative time

**Fig. 2:**
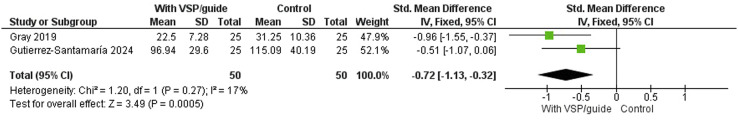
Forest plot for operative time.

Forest plots in Fig. 2 demonstrate that operative time was significantly shorter with VSP guidance compared with conventional methods without VSP (SMD = −0.72; 95 % CI −1.13 to −0.32; P < 0.05).

### Accuracy

One study with a comparator reported diagnostic accuracy of 95.25 ± 4.09 % with VSP versus 78.25 ± 12.92 % without. Another study without a comparator reported 89.93 ± 8.6 %. Meta-analysis was not feasible due to limited comparative data.

### Safety and complications

One study with a comparator reported safety rates of 95.25 ± 4.09 % with VSP versus 78.25 ± 12.92 % without. Two additional studies without comparators reported very low complication rates of 1.77 % and 0 %. Meta-analysis was not feasible, and definitive conclusions regarding safety cannot be drawn, given the lack of comparative analysis of bias in outcome reporting present in the available studies.

### Satisfaction, perceived femininity, and cosmetic outcomes

Gutierrez-Santamaría et al. described the results of bespoke satisfactions scale which assessed satisfaction on a 10-point Likert scale (On a scale of 1 to 10, with 1 being “totally unsatisfied” and 10 “totally satisfied”).^18^ LaPadula et al. used the Satisfaction with Life Scale (SWLS) and Subjective Happiness Scale (SHS), both validated measures using 7-point Likert scales, which higher scores representation higher satisfaction with life or happiness respectively).^20^^,^^22^, ^23^ Tawa et al. described the results of a bespoke, 5-point satisfaction survey, administered anonymously by a surgical resident to participants, using a 5-point Likert scale assessing agreement with questions considering satisfaction of post-operative outcome.^21^ Given assessment of satisfaction outcomes using heterogeneous tools, with no study included a comparator group, pooled analysis was not feasible. Despite this, satisfaction and cosmetic outcomes were generally reported as high (Table 3).

## Discussion

This systematic review synthesizes the available evidence regarding the role of VSP in osseous FFS. The findings of available papers included in this review demonstrated benefit with use of VSP for guiding intraoperative osteotomy for operative efficiency, accuracy, safety, and patient-reported outcomes including measures of post-operative satisfaction. Although the quantity and quality of comparative evidence remain limited, with heterogeneity of available literature limiting the feasible of meta-analysis for included study data, the collective findings support an expanding role for VSP and 3D reconstruction assisted navigation, in aiding preoperative planning and intraoperative precision, facilitating overall workflow optimization in craniofacial gender-affirming surgery.

Operative time was demonstrated to be significantly reduced with VSP guided procedures compared to traditional techniques, on average, 13.45 min across procedures reporting this outcome. Even in studies without comparators, streamlined intraoperative execution was a recurring theme. This finding is supported by the results of Andrew et al., who suggested a role for VSP in reducing operative time in other craniomaxillofacial procedures, with VSP significantly reducing operative time in both single suture and multiple suture craniosynostosis repair. Moreover, operative time decreased further with increasing surgeon familiarity with VSP technology, highlighting the compounding benefits VSP may offer.^24^ These findings are also supported by the results of the review by Mavrommatis and colleagues, who suggested improvements in surgical accuracy and decreases in operative times for craniofacial and head and neck reconstructive surgery with the use of VSP.^25^ In osseous FFS, reductions in operative time may reflect improved preoperative mapping, streamlined intraoperative decision making, and enhanced execution via cutting guides or custom implants.^24^ Efficiency gains in such procedures offer multi-level benefit, where reduced anaesthetic time can reduce perioperative complications and length of stage, and improved theatre utilisation can lead to economic benefits.

Considering cost-effectiveness of VSP for FFS, while there was limited quantitative data available to provide a comprehensive cost-effectiveness evaluation, enhanced intraoperative accuracy and reduced operative time offers may offset the upfront costs of VSP technology, aided in the long term by with surgeon learning curve and increased software familiarity. Further utility assessment is required before definitive economic considerations can be made, however reduced operative times offers promise considering the long-term financial benefits of VSP overall. While the financial overheads of VSP are typically underreported in the literature, the available evidence suggests the cost of using commercially provided VSP is between 3000–5000 USD per case, up to 9000 when surgical cutting guides are included. Costs typically relate to software licensing, cutting guide fabrication, in house point of care printing, and full custom patient specific implants.^26^ As the hospital are typically responsible for the upfront costs of procedures, the overall cost benefits must be weighed when considering procedure demand and hardware utilisation for overall estimation of benefit.

Planning time varied substantially in available literature. This heterogeneity likely reflects differences in software platforms, workflow integration, in-house versus outsourced planning, and whether custom guides or implants were fabricated. Importantly, planning time must be contextualised within the broader perioperative pathway: increased preoperative digital preparation may be offset by intraoperative time savings and improved predictability of osteotomy and overall surgical outcomes. Ostas and colleagues described in their review of VSP and 3D printing in oral and craniomaxillofacial surgery how planning time may vary depending on the scope and complexity of intervention and planning software used; moreover, highlighting how professional software may significantly reduce planning time due to reductions in learning curves and increased software usability.^27^ Overall, planning time is inherently linked to complexity of the intervention and software capabilities; hence findings should be contextualised in patient and procedure specific demographic and clinical factors.

Accuracy outcomes were in support of the use of VSP, with findings of both Gray et al. and Tawa et al. suggesting a marked benefit of VSP in improving accuracy of custom guides in FFS procedures. These results align with broader craniofacial literature demonstrating improved conformity between planned and achieved osteotomies when digital planning and guide-based execution are used. Lee, Oh, and Kim (2024) described how VSP, particularly when integrated with patient specific osteotomy guides may significantly reduce the discrepancy between planning and actual surgical outcomes in orthognathic surgery, highlighting VSPs evolving role in improving surgical precision. Their study highlighted how the influence on operative time was not conclusively significant however, highlighting where further research is required to make definition conclusions regarding the range of potential benefits of VSP, and overall complexity of outcome assessment.^28^

Considering the cost-effectiveness of VSP for use in FFS procedures, the applicability of VSP to the range of FFS procedures cane be considered, where the upfront cost of technological and increased complexity of operative steps is worth the enhanced accuracy offered by support of intraoperative navigation with VSP. In frontal sinus reduction, the variability of the frontal sinus anatomy requires high accuracy of preoperative templating and planning of osteotomies; moreover, the posterior table and proximity of intracranial anatomy mean that high levels of accuracy are essential to optimise outcomes, hence here VSP may offer added value.^15^ In genioplasty, the three-dimensional nature of the intraoperative navigation required, means that precise osteotomy level planning with VSP support the simultaneous adequate addressal of anterior projection, vertical height, width and transverse symmetry; hence VSP offers clear benefit.^17^^,^^28^ Other procedures such as mandibular angle reduction and orbital rim contouring offer a less clearly defined indication for VSP, where the upfront costs may be considered insufficient to justify use where surgeon expertise can provide satisfactory outcomes.^29^

In the context of FFS, where subtle contour changes influence perceived gender congruence, enhanced perioperative osteotomy accuracy is of significant importance. Precise frontal sinus setback, orbital contouring, and mandibular angle reduction are critical to achieving the aesthetically desired feminization of the skeletal framework while avoiding functional compromise.^29^ This has subsequent influence on patient outcomes relating to gender incongruence, highlighting the potential role of VSP and importance of accurately characterizing its role in this patient specific subgroup.

Safety data were limited but encouraging, with included studies suggesting low complication and high safety rates. The absence of robust comparator groups and limited follow-up constrain definitive conclusions in this subspeciality field at present. Tel et al., 2024 describe the role of VSP in guiding complex craniofacial oncologic resections, where real-time anatomical visualization, spatial orientation, and cognitive guidance intraoperatively, enhanced surgical precision, via improved anatomical navigation.^30^ This has translational relevance to FFS, where given the proximity of osteotomies to the frontal sinus, dura, and neurovascular structures, enhanced anatomical mapping through VSP reduces risk of unintentional dural exposure or breach or nerve injury.

Across studies, satisfaction and cosmetic outcomes were high. However, heterogeneous assessment tools (bespoke Likert scales versus validated instruments such as SWLS and SHS) precluded pooled analysis. Despite this limitation, the consistently positive trend suggests that enhanced precision and preoperative visualisation may contribute to improved patient confidence and perceived femininity and warrant further investigation with validated patient-reported outcome measures. The GENDER-Q is a recently validated patient reported outcome measure for outcomes of gender-affirming care, including facial feminizing surgery; assessment of the influence of surgery on quality of life and satisfaction with FFS specific aesthetic and functional outcomes using this measure may provide more tangible evidence of the perioperative benefits of procedures on health-related quality of life.^31^

### Limitations

The principal limitation of this review lies in the heterogeneity of available literature. Included studies varied widely in study design, being mostly retrospective cohorts and case series, sample size and procedural mix, varying significantly in definitions and measurement of outcomes, and reporting of confounders. Six of seven studies demonstrated moderate risk of bias, most commonly due to confounding and incomplete methodological reporting. Only one outcome (operative time) permitted quantitative synthesis. Accuracy, safety, and satisfaction data were inconsistently defined and reported, precluding robust meta-analytic comparison. Additionally, the rapid evolution of digital surgical technology introduces temporal heterogeneity; earlier VSP systems differ substantially from contemporary platforms incorporating artificial intelligence–assisted segmentation, in-house 3D printing, and emerging AR overlays. Cost analysis was inconsistently addressed, limiting conclusions regarding economic value.

These limitations highlight the early and emerging role of VSP in guiding precision osteotomy in FFS. Further randomized, matched comparative prospective studies will provide more tangible evidence of benefits and superiority of VSP guided procedures to traditional FFS, although this early evidence is favourable in support of this emerging role, although directional trends are favourable.

## Conclusion

Despite heterogeneity and methodological limitations, the findings of this review demonstrate consistent trends toward reduced operative time, improved accuracy, high safety, and strong patient satisfaction with VSP-guided osseous FFS. While definitive comparative conclusions are not feasible with the current evidence base, the existing available data supports VSP as an increasingly evolving tool for modern FFS. Innovation now extends toward AR-assisted intraoperative navigation, which may further enhance precision, safety, and aesthetic predictability as the next translation step with real-time projection of preoperative VSP plans onto the operative field. In facial plastic surgery more broadly, AR and mixed-reality systems have demonstrated feasibility for intraoperative navigation and enhanced anatomical orientation.^32^ In craniofacial oncology, AR guidance has been successfully used to execute complex resections with high spatial fidelity.^30^ The integration of AR-VSP into FFS will facilitate verification of osteotomy limits in real time, enhance the mapping of frontal sinus limits and supraorbital contour, improve assessment of osteotomy symmetry, and reduce reliance on static cutting guides alone. Given the aesthetic precision demanded in FFS and the psychosocial importance of outcome, AR-assisted VSP and intraoperative navigation may become particularly valuable, in particularly in cases of revision surgery or anatomical complex individual patients or procedures. To consolidate the role of VSP and AR in FFS, future research should prioritise prospective comparative studies with standardised outcome measures, adequate patient-reported outcome assessment using validated and population specific outcome measures, and long-term follow-up, assessing revision rates and durability of contour outcomes. Cost-effectiveness analyses are also key, with adequate assessment of savings relating to operative time, complication reduction, and patient-reported quality of life.

## Registration and protocol

This systematic review was conducted in accordance with the Cochrane handbook for systematic reviews and has been reported in line with the Preferred Reporting Items for Systematic Reviews and Meta-Analyses (PRISMA) statement. The protocol for this review was prospectively registered with PROSPERO (ID:).

## Funding

No financial support or funding was received for this study.

## Data availability

The data used in this review is provided in the manuscript.

## Ethical approval

Not required.

## Declaration of competing interest

The authors wish to declare that there are no conflicts of interest.

## Appendix Supplementary materials

Appendix A Full search strategy Image, application 1

Appendix B Risk of bias assessment Image, application 2

## References

[1] Singh G.D., Singh M. (2021). Virtual Surgical planning: modeling from the present to the future. J Clin Med.

[2] González-López P., Kuptsov A., Gómez-Revuelta C., Fernández-Villa J., Abarca-Olivas J., Daniel R.T., Meling T.R., Nieto-Navarro J. (2024). The integration of 3D virtual reality and 3D printing technology as innovative approaches to preoperative planning in neuro-oncology. J Pers Med.

[3] Telang P.S. (2020). Facial feminization surgery: a review of 220 consecutive patients. Indian J Plast Surg.

[4] Raner G.A., Jaszkul K.M., Bonapace-Potvin M., Al-Ghanim K., Bouhadana G., Roy A.A., Bensimon É (2024). Quality of life outcomes in patients undergoing facial gender affirming surgery: a systematic review and meta-analysis. Int J Transgend Health.

[5] Altman K. (2012). Facial feminization surgery: current state of the art. Int J Oral Maxillofac Surg.

[6] Lundgren T.K., Farnebo F. (2017). Midface osteotomies for feminization of the facial skeleton. Plast Reconstr Surg Glob Open.

[7] Spiegel J.H. (2019). Discussion: osseous transformation with facial feminization surgery: improved anatomical accuracy with virtual planning. Plast Reconstr Surg.

[8] Escandón J.M., Morrison C.S., Langstein H.N., Ciudad P., Del Corral G., Manrique O.J. (2022). Applications of three-dimensional surgical planning in facial feminization surgery: a systematic review. J Plast Reconstr Aesthet Surg.

[9] Louis M., Qiu C.S., Travieso R., Marano D., Coon D. (2022). Computer-aided planning and execution in facial gender surgery: approaches, concepts, and implementation. Plast Reconstr Surg Glob Open.

[10] Sharaf B., Kuruoglu D., Bite U., Morris J.M. (2022). Point of care virtual surgical planning and 3D printing in facial feminization surgery. Semin Plast Surg.

[11] Covidence systematic review software, Veritas Health Innovation, Melbourne, Australia. Available at www.covidence.org.

[12] Review Manager (RevMan) [computer program]. Version 7.12.0. The Cochrane Collaboration; 2024. Available from: revman.cochrane.org

[13] Higgins J.P.T., Thomas J., Chandler J., Cumpston M., Li T., Page M.J. (2024). Cochrane Handbook for Systematic Reviews of Interventions.

[14] Sterne J.A., Hernán M.A., Reeves B.C., Savović J., Berkman N.D., Viswanathan M., Henry D., Altman D.G., Ansari M.T., Boutron I., Carpenter J.R., Chan A.W., Churchill R., Deeks J.J., Hróbjartsson A., Kirkham J., Jüni P., Loke Y.K., Pigott T.D., Ramsay C.R., Regidor D., Rothstein H.R., Sandhu L., Santaguida P.L., Schünemann H.J., Shea B., Shrier I., Tugwell P., Turner L., Valentine J.C., Waddington H., Waters E., Wells G.A., Whiting P.F., Higgins J.P. (2016). ROBINS-I: a tool for assessing risk of bias in non-randomised studies of interventions. BMJ.

[15] Ganry L., Cömert M. (2022). Low-cost and simple frontal sinus surgical cutting guide modeling for anterior cranioplasty in facial feminization surgery: how to do it. J Craniofacial Surg.

[16] Gray R., Nguyen K., Lee J.C., Deschamps-Braly J., Bastidas N., Tanna N., Bradley J.P. (2019). Osseous transformation with facial feminization surgery: improved anatomical accuracy with virtual planning. Plast Reconstr Surg.

[17] Gursky A.K., Chinta S.R., Wyatt H.P., Belisario M.N., Shah A.R., Kantar R.S., Rodriguez E.D. (2024). A comprehensive analysis of genioplasty in facial feminization surgery: a systematic review and institutional cohort study. J Clin Med.

[18] Gutiérrez-Santamaría J., Simon D., Capitán L., Bailón C., Bellinga R.J., Tenório T., Sánchez-García A., Capitán-Cañadas F. (2024). Shaping the lower jaw border with customized cutting guides: development, validation, and application in facial gender-affirming surgery. Facial Plast Surg Aesthet Med.

[19] Hu S., Lawrence J., Schuster C.R., Gunduz Sarioglu A., Yusuf C., Reiche E., Parisi M.N., Rahmayanti S., Bommineni V., Soares V., Yang R., Coon D. (2025). Moving to 3D: quantifying virtual surgical planning accuracy using geometric morphometrics and cephalometrics in facial feminization surgery. J Craniofacial Surg.

[20] La Padula S., Hersant B., Chatel H., Aguilar P., Bosc R., Roccaro G., Ruiz R., Meningaud J.P. (2019). One-step facial feminization surgery: the importance of a custom-made preoperative planning and patient satisfaction assessment. J Plast Reconstr Aesthet Surg.

[21] Tawa P., Brault N., Luca-Pozner V., Ganry L., Chebbi G., Atlan M., Qassemyar Q. (2021). Three-dimensional custom-made surgical guides in facial feminization surgery: prospective study on safety and accuracy. Aesthet Surg J..

[22] Diener E., Emmons R.A., Larsen R.J., Griffin S. (1985). The satisfaction with life scale. J Pers Assess.

[23] Lyubomirsky S., Lepper H.S. (1999). A measure of subjective happiness: preliminary reliability and construct validation. Soc Indic Res.

[24] Andrew T.W., Baylan J., Mittermiller P.A., Cheng H., Johns D.N., Edwards M.S.B., Cheshier S.H., Grant G.A., Lorenz H.P. (2018). Virtual surgical planning decreases operative time for isolated single suture and multi-suture craniosynostosis repair. Plast Reconstr Surg Glob Open.

[25] Mavrommatis M.A., Liu K., Wilson J. (2025). Virtual surgical planning in facial gender affirming surgery. Curr Otorhinolaryngol Rep.

[26] Lawless M., Swendseid B., WindheimN V.O.N., VanKoevering K., Seim N., Old M. (2022). Review of cost and surgical time implications using virtual patient specific planning and patient specific implants in midface reconstruction. Plast Aesthet Res.

[27] Ostaș D., Almășan O., Ileșan R.R., Andrei V., Thieringer F.M., Hedeșiu M., Rotar H. (2022). Point-of-care Virtual surgical planning and 3D printing in oral and cranio-maxillofacial surgery: a narrative review. J Clin Med.

[28] Lee Y.J., Oh J.H., Kim S.G. (2024). Virtual surgical plan with custom surgical guide for orthognathic surgery: systematic review and meta-analysis. Maxillofac Plast Reconstr Surg.

[29] Borbon C., Novaresio A., Iocca O., Nonis F., Moos S., Vezzetti E., Ramieri G., Zavattero E. (2025). Evaluating osteotomy accuracy in mandibular reconstruction: a preliminary study using custom cutting guides and virtual reality. Diseases.

[30] Tel A., Raccampo L., Vinayahalingam S., Troise S., Abbate V., Orabona G.D., Sembronio S., Robiony M. (2024). Complex craniofacial cases through augmented reality guidance in surgical oncology: a technical report. Diagnostics (Basel).

[31] Kaur M.N., Rae C., Morrison S.D. (2025). Development and assessment of a patient-reported outcome instrument for gender-affirming care. JAMA Netw Open.

[32] Chou D.W., Annadata V., Willson G., Gray M., Rosenberg J. (2024). Augmented and virtual reality applications in facial plastic surgery: a scoping review. Laryngoscope.

