# Supplementary material for: Virtual surgical planning guided osteotomy in facial feminization surgery
Source: JPRAS Open. 2026 Jun 3;50:699–711. doi: 10.1016/j.jpra.2026.05.039 (PMC13312027; doi:10.1016/j.jpra.2026.05.039)
Supplement: Supplementary file 1 — Appendix A Full search strategy [file mmc1.docx]

Appendix 1 Search strategy for each data source using Boolean Operators

| Database | Boolean operator |
| --- | --- |
| Medline (via Ovid) | 1. virtual surgical planning.ab,kf,kw,ti.  2. VSP.ab,kf,kw,ti.  3. guided surgery.ab,kf,kw,ti.  4. surgical guidance.ab,kf,kw,ti.  5. virtual reality.ab,kf,kw,ti.  6. VR.ab,kf,kw,ti.  7. 3D surgical planning.ab,kf,kw,ti.  8. Three-Dimensional surgical planning.ab,kf,kw,ti.  9. computer assisted surgery.ab,kf,kw,ti.  10. *surgery, computer-assisted/  11. virtual planning.ab,kf,kw,ti.  12. facial feminization.ab,kf,kw,ti.  13. facial feminization surgery.ab,kf,kw,ti.  14. FFS.ab,kf,kw,ti.  15. facial gender surgery.ab,kf,kw,ti.  16. gender affirming facial surgery.ab,kf,kw,ti.  17. gender reassignment procedure.ab,kf,kw,ti.  18. gender confirming facial surgery.ab,kf,kw,ti.  19. forehead feminization.ab,kf,kw,ti.  20. frontal sinus setback.ab,kf,kw,ti.  21. feminization rhinoplasty.ab,kf,kw,ti.  22. chin feminization.ab,kf,kw,ti.  23. genioplasty feminization.ab,kf,kw,ti.  24. lower jaw feminization.ab,kf,kw,ti.  25. mandibular feminization.ab,kf,kw,ti.  26. jaw reduction feminization.ab,kf,kw,ti.  27. transgender.ab,kf,kw,ti.  28. 1 or 2 or 3 or 4 or 5 or 6 or 7 or 8 or 9 or 10 or 11  29. 12 or 13 or 14 or 15 or 16 or 17 or 18 or 19 or 20 or 21 or 22 or 23 or 24 or 25 or 26 or 27  30. 28 and 29 |
| Embase | #1 'virtual surgical planning':ti,ab,kw OR 'guiding device':ti,ab,kw OR 'virtual reality':ti,ab,kw OR 'virtual reality system':ti,ab,kw OR 'computer analysis':ti,ab,kw  #2. 'facial feminization surgery':ti,ab,kw OR 'facial feminization':ti,ab,kw OR 'gender reassignment':ti,ab,kw OR 'gender affirming surgery':ti,ab,kw OR 'feminization':ti,ab,kw OR 'feminization surgery':ti,ab,kw OR 'frontal sinus surgery':ti,ab,kw OR 'genioplasty':ti,ab,kw OR 'transgender':ti,ab,kw OR (transgender:ti,ab,kw AND 'gender nonbinary':ti,ab,kw) OR transgender:ti,ab,kw  #3. #1 AND #2 |
| Cochrane Library | #1 (virtual surgical planning):ti,ab,kw OR (VSP):ti,ab,kw OR (guided surgery):ti,ab,kw OR (surgical guidance):ti,ab,kw OR (virtual reality):ti,ab,kw  #2 (VR):ti,ab,kw OR (3D surgical planning):ti,ab,kw OR (three-dimensional surgical planning):ti,ab,kw OR ("computer assisted surgery"):ti,ab,kw OR (virtual planning):ti,ab,kw  #3 #1 OR #2  #4 (facial feminization):ti,ab,kw OR (facial feminization surgery):ti,ab,kw OR (FFS):ti,ab,kw OR (facial gender surgery):ti,ab,kw OR (gender affirming surgery):ti,ab,kw  #5 (gender reassignment surgery):ti,ab,kw OR (gender confirming surgery):ti,ab,kw OR (forehead feminization):ti,ab,kw OR (frontal sinus setback):ti,ab,kw OR (feminization rhinoplasty):ti,ab,kw  #6 (chin feminization):ti,ab,kw OR (genioplasty feminization):ti,ab,kw OR (chin feminization):ti,ab,kw OR (lower jaw feminization):ti,ab,kw OR (mandibular feminization):ti,ab,kw  #7 (jaw reduction feminization):ti,ab,kw OR (transgender):ti,ab,kw  #8 #4 OR #5 OR #6 OR #7  #9 #3 AND #8 |
| Scopus | ( TITLE-ABS-KEY ( virtual AND surgical AND planning ) OR TITLE-ABS-KEY ( vsp ) OR TITLE-ABS-KEY ( guided AND surgery ) OR TITLE-ABS-KEY ( surgical AND guidance ) OR TITLE-ABS-KEY ( virtual AND reality ) OR TITLE-ABS-KEY ( vr ) OR TITLE-ABS-KEY ( 3d AND surgical AND planning ) OR TITLE-ABS-KEY ( three AND dimensional AND surgical AND planning ) OR TITLE-ABS-KEY ( computer AND assisted AND surgery ) OR TITLE-ABS-KEY ( virtual AND planning ) ) AND ( TITLE-ABS-KEY ( facial AND feminization ) OR TITLE-ABS-KEY ( ffs ) OR TITLE-ABS-KEY ( facial AND gender AND surgery ) OR TITLE-ABS-KEY ( gender AND affirming AND facial AND surgery ) OR TITLE-ABS-KEY ( gender AND reassignment AND procedure ) OR TITLE-ABS-KEY ( gender AND confirming AND facial AND surgery ) OR TITLE-ABS-KEY ( forehead AND feminization ) OR TITLE-ABS-KEY ( frontal AND sinus AND setback ) OR TITLE-ABS-KEY ( facial AND feminization AND surgery ) OR TITLE-ABS-KEY ( feminization AND rhinoplasty ) OR TITLE-ABS-KEY ( chin AND feminization ) OR TITLE-ABS-KEY ( genioplasty ) OR TITLE-ABS-KEY ( lower AND jaw AND feminization ) OR TITLE-ABS-KEY ( mandibular AND feminization ) OR TITLE-ABS-KEY ( jaw AND reduction AND feminization ) OR TITLE-ABS-KEY ( transgender ) ) |
| References from related articles | Manual search |
