# Supplementary figures and images for: Virtual surgical planning guided osteotomy in facial feminization surgery
Source: JPRAS Open. 2026 Jun 3;50:699–711. doi: 10.1016/j.jpra.2026.05.039 (PMC13312027; doi:10.1016/j.jpra.2026.05.039)

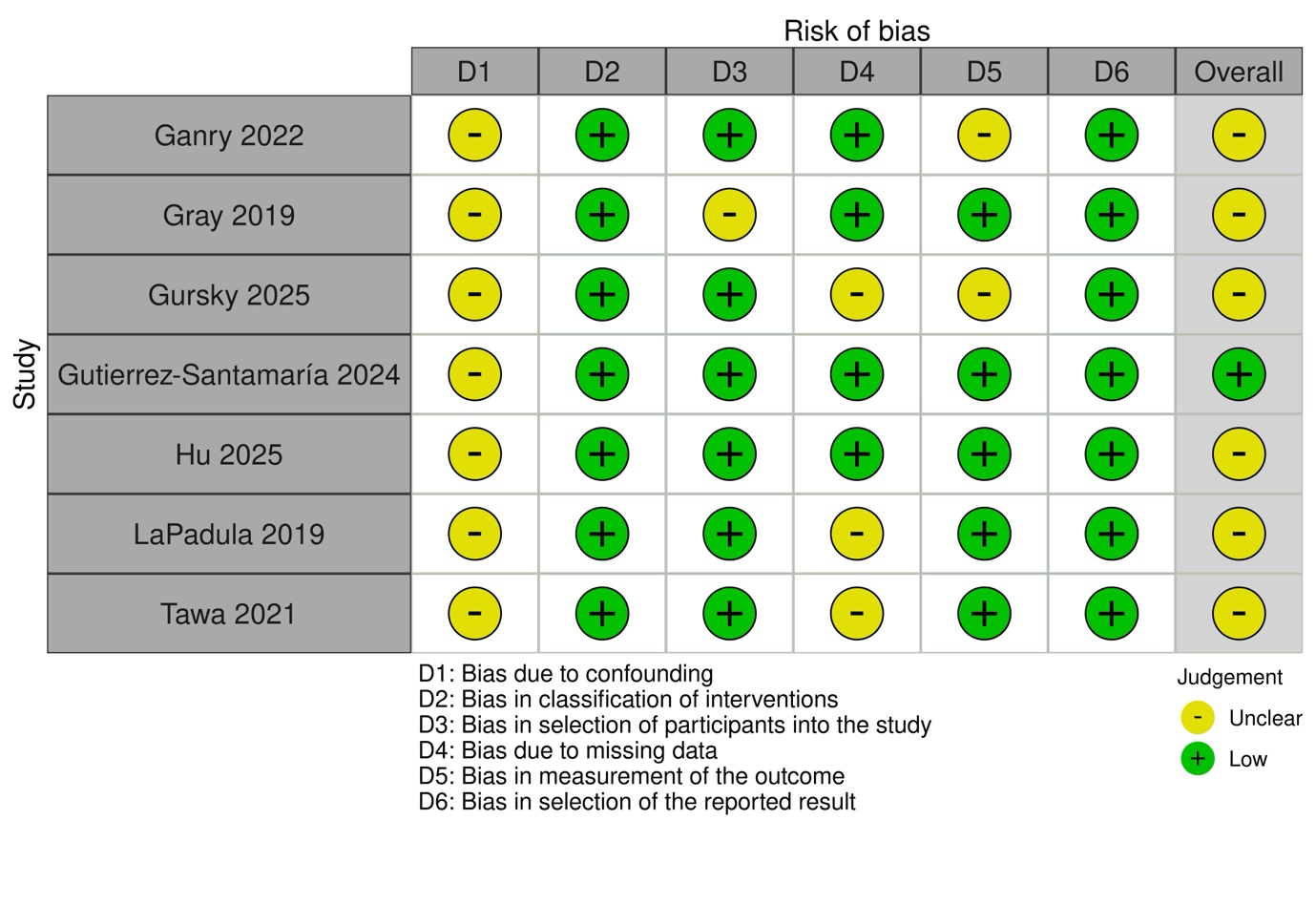

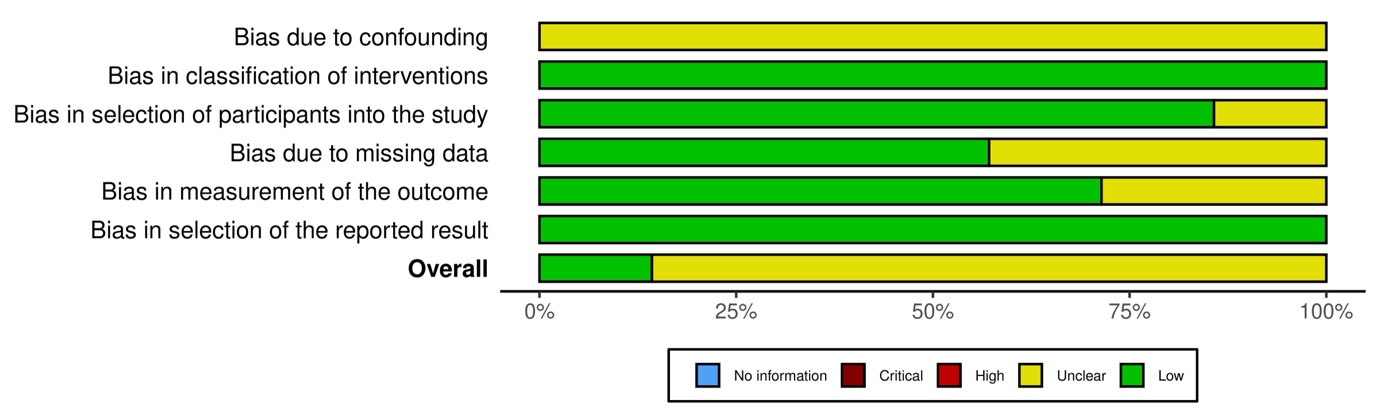

Supplement: Supplementary file 2 — Appendix B Risk of bias assessment [file mmc2.docx]
